# Infective Endocarditis–Associated Glomerulonephritis With Cryoglobulin Positivity

**DOI:** 10.1016/j.jaccas.2025.104048

**Published:** 2025-07-16

**Authors:** Kenta Uemura, Norio Kanamori, Takeshi Aoyama

**Affiliations:** Division of Cardiology, Shimada General Medical Center, Shimada, Japan

**Keywords:** autoimmune, endocarditis, immunization, mitral valve

## Abstract

**Background:**

Serum cryoglobulins are frequently elevated in cases of infective endocarditis–associated glomerulonephritis (IEAGN). We report a case of cryoglobulin-positive IEAGN, initially diagnosed as cryoglobulinemic glomerulonephritis.

**Case Summary:**

A woman in her 70s visited our hospital with a rapidly declining renal function. Cryoglobulins were detected in her blood sample, and findings of the renal biopsy initially indicated cryoglobulinemic glomerulonephritis. Despite initiating steroid therapy, her renal function did not improve. Further investigations, including an echocardiogram, revealed mitral valve vegetation, and blood cultures identified a gram-positive coccus. These findings led to a revised diagnosis of IEAGN. After the initiation of antimicrobial therapy, the patient’s serum cryoglobulin levels normalized, and her renal function improved.

**Discussion:**

Cryoglobulinemia is frequently observed in cases of IEAGN, but these cryoglobulins are often clinically insignificant and tend to resolve with appropriate antimicrobial therapy.

**Take-Home Message:**

In the case of cryoglobulin-positive nephritis, the possibility of IEAGN should always be considered, requiring careful differentiation from other cryoglobulin-associated diseases.

## Visual Summary

**Figure:**
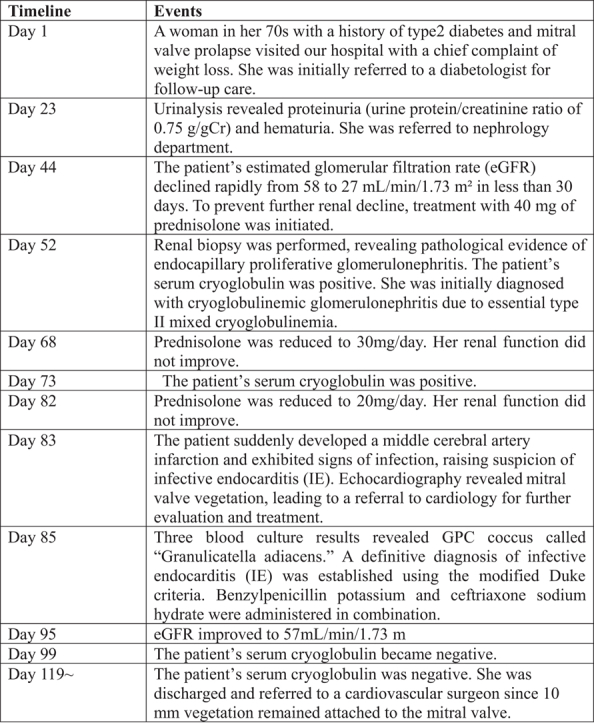

**Take-Home Message**

• In case of cryoglobulin-positive nephritis, the possibility of infective endocarditis-associated glomerulonephritis should always be considered, requiring careful differentiation from other cryoglobulin-associated diseases.

## History of Presentation

A woman in her 70s with a history of diabetes and and mitral valve prolapse visited our hospital with a primary concern of weight loss. She had a low-grade fever of 37.5 °C but reported no other specific symptoms.

She was initially referred to a diabetologist for follow-up care, where urinalysis revealed proteinuria (urine protein/creatinine ratio of 0.75 g/gCr) and hematuria. Over 30 days, her estimated glomerular filtration rate declined rapidly from 58 to 27 mL/min/1.73 m^2^, prompting referral to a nephrologist.

The patient was initially diagnosed with cryoglobulinemic glomerulonephritis due to essential type II mixed cryoglobulinemia. To prevent further renal decline, treatment with 40 mg of prednisolone was initiated.

Despite 3 weeks of steroid therapy and subsequent tapering, her renal function did not improve. During her hospitalization, she developed a middle cerebral artery infarction and exhibited signs of infection, raising suspicion of infective endocarditis (IE). Transthoracic echocardiography (TTE) revealed mitral valve vegetation, prompting a referral to a cardiologist for further evaluation and treatment.

## Past Medical History

The patient had a history of type 2 diabetes, dyslipidemia, mitral regurgitation, and angina, and she was previously treated with percutaneous coronary intervention for the latter conditions. Although she had mitral regurgitation associated with mitral valve prolapse for 8 years, TTE performed 2 years before the admission showed no evidence of vegetations (Video 1). Her medications included HMG-CoA reductase inhibitors, metformin hydrochloride, aspirin, and clopidogrel. Her family history was unremarkable, and she had no notable lifestyle habits, such as smoking or alcohol consumption.

## Differential Diagnosis

Clinical manifestations are consistent with cryoglobulinemic glomerulonephritis. Possible etiologic causes of cryoglobulin positivity include collagen disease, hepatitis C virus (HCV) infection, bacterial infection, and lymphoma. Antineutrophil cytoplasmic antibody (ANCA)–associated vasculitis and IgA nephropathy were also considered in the context of impaired renal function.

## Investigations

The patient was alert and ambulatory at the time of presentation. She had a low-grade fever of approximately 37.5 °C, a known history of mitral regurgitation, and a holosystolic murmur. No immunologic phenomena, such as Osler nodes or Janeway lesions, were observed. Electrocardiogram and chest radiograph showed no abnormalities.

Laboratory test results revealed anemia (hemoglobin: 6.0 g/dL), impaired renal function (serum creatinine: 1.51 mg/dL, estimated glomerular filtration rate: 27 mL/min/1.73 m^2^), and a mildly elevated inflammatory marker (C-reactive protein: 3.99 mg/dL). Serum IgG levels were elevated (2,238 mg/dL), and proteinase 3-ANCA levels were mildly elevated (26.1 IU/mL). Complement levels were decreased, but antinuclear antibodies were negative, with no findings suggesting an autoimmune disease. Cryoglobulins were detected as immune complexes in the precipitate consisting of monoclonal IgM and polyclonal IgG.

Upper gastrointestinal endoscopy, bone marrow biopsy, and computed tomography ruled out hematologic or malignant disease.

**Figure 1:**
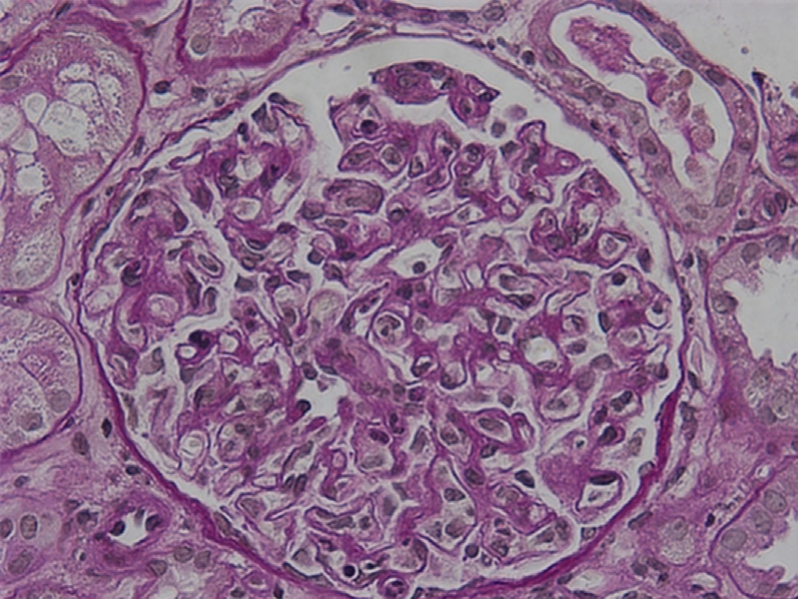
A Periodic Acid–Schiff Stain of Glomerulus Sixteen glomeruli were found on a single renal biopsy, and histologic findings were endoproliferative glomerulonephritis. No evidence of crescent formation, necrotizing lesions, and periodic acid–Schiff stain–positive cryoplugs was noted.

**Figure 2:**
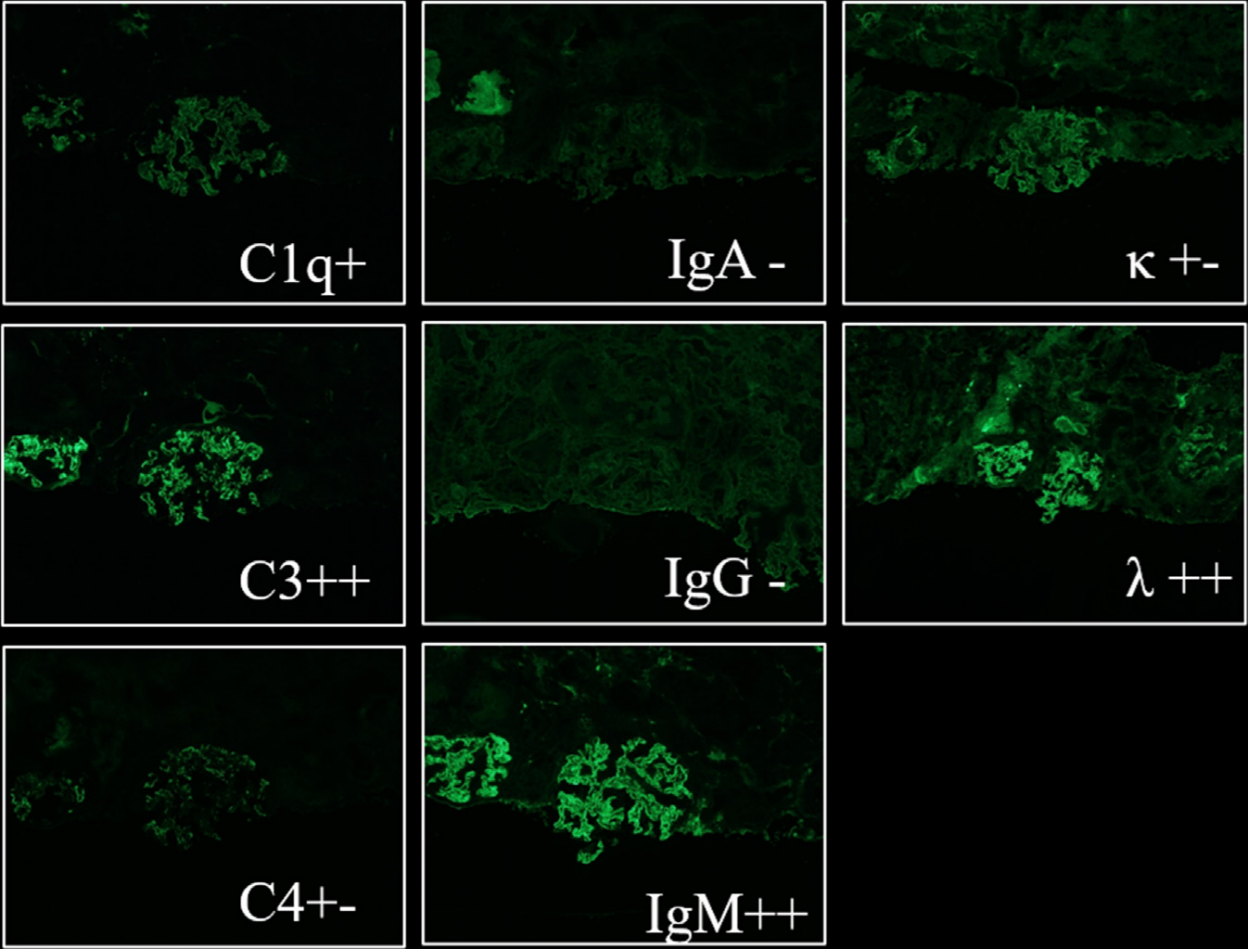
Immunofluorescence Staining of Glomerulus IgM, C3, and C1q were positive.

A renal biopsy was performed a few days after the initiation of prednisolone therapy, revealing pathologic evidence of endocapillary proliferative glomerulonephritis. No evidence of crescent formation, necrotizing lesions, or periodic acid–Schiff stain–positive cryoplugs was observed in any of the 16 identified glomeruli (Figure 1). The immunofluorescence stain showed positive results for IgM, C3, and C1q (Figure 2). Electron microscopy could not be performed because of equipment limitations at our facility. TTE identified an 11-mm vegetation on the posterior leaflet of the mitral valve (Video 2). Transesophageal echocardiography was not performed throughout the inspection.

Three sets of blood cultures were positive for *Granulicatella adiacens*, a gram-positive coccus. Based on the initial findings, cryoglobulinemic glomerulonephritis was suspected. Although cryoglobulinemia is often associated with viral infections (eg, HCV), autoimmune diseases, or hematologic conditions such as malignant lymphoma, none of these were true in this case.

Although bacterial infections can also cause cryoglobulinemia, blood cultures were not performed during the initial clinical evaluation because there were no symptoms suggestive of bacterial infection, and the mitral valve vegetation was considered a previously known mitral valve prolapse. Consequently, the patient was diagnosed with essential type II mixed cryoglobulinemia with cryoglobulinemic glomerulonephritis, under the assumption that no underlying disease was present. However, the ineffectiveness of immunosuppressive therapy prompted a revaluation of the diagnosis. On reviewing the pathologic findings, the absence of periodic acid–Schiff stain–positive cryoplugs—typical markers of cryoglobulinemic glomerulonephritis—raised doubt about the initial diagnosis.

Although the patient’s blood sample was positive for ANCA, ANCA-associated nephritis was deemed unlikely because of the absence of pauci-immune necrotizing crescentic glomerulonephritis on the pathologic images. The diagnosis shifted when blood culture results proved bacteremia. A definitive diagnosis of IE was established using the modified Duke criteria^1^: the patient had 1 major criterion (echocardiographic evidence of mitral valve vegetation) and 3 minor criteria (a predisposing heart condition of mitral regurgitation, vascular phenomena including cerebral infarction, and a history of intravenous drug use before presentation), fulfilling the criteria for a definitive diagnosis of IE. In addition, the presence of hypocomplementemia and C3- and IgM-dominant immune deposits on immunofluorescence staining supported a diagnosis of infective endocarditis–associated glomerulonephritis (IEAGN). Ultimately, the patient was diagnosed with cryoglobulin-positive IEAGN.

## Management

Benzylpenicillin potassium and ceftriaxone sodium hydrate were administered in combination to the patient, following the recommendations outlined in the 2023 European Society of Cardiology guidelines for the management of IE.^2^

Although *G adiacens* was an unfamiliar pathogen, we referred to a previously published case report documenting successful treatment of IE caused by *G adiacens*,^3^ which guided our treatment approach.

## Outcome and Follow-Up

The patient’s renal function began to improve soon after the initiation of antimicrobial therapy, returning to baseline levels within 2 weeks. Serum cryoglobulin tests turned negative 15 days after starting treatment. All her symptoms resolved within 4 weeks, and she was discharged without any lasting complications.

However, a 10-mm vegetation remained attached to the mitral valve and necessitated surgical removal, prompting a referral to a cardiovascular surgeon.

After the surgery, the patient continues to attend regular follow-up appointments at our hospital with no recurrence of IE.

## Discussion

Glomerulonephritis caused by IE is defined as IEAGN, and elevated serum cryoglobulins are noted in some cases. Although the exact prevalence of cryoglobulin positivity in IEAGN remains unclear, one article suggests that up to 90% of patients with IE may be positive for cryoglobulin.^4^ Another study reports that 47% of ANCA-positive infection-related glomerulonephritis cases are also cryoglobulin positive.^5^ Although the reported frequency and definition of cryoglobulin positivity vary across the literature, it is widely accepted that cryoglobulinemia is commonly associated with IEAGN.

However, not all serum cryoglobulin is clinically significant. In our case, cryoglobulinemia is not present as cryoglobulin vasculitis. Nonetheless, there have been reported cases of IE-induced cryoglobulinemia progressing to cryoglobulinemic vasculitis.^6^^,^^7^ The likelihood of cryoglobulinemia developing into cryoglobulinemic vasculitis is uncertain but considered low, with estimates ranging from 12.3% to 27% in HCV-induced cryoglobulinemia cases.^8^ In IEAGN, the presence of serum cryoglobulin is often clinically insignificant unless renal biopsy findings indicate cryoglobulin involvement contributing to renal dysfunction.

Differentiating cryoglobulin-positive IEAGN from other cryoglobulin-related diseases is crucial because misdiagnoses have been reported in similar cases.^9^ Because antimicrobial therapy and immunosuppressive therapy are fundamentally opposite, it is important to thoroughly investigate the underlying etiology before initiating treatment.

Although our patient achieved remission with antimicrobial therapy, the overall prognosis for renal function recovery in IEAGN is generally poor, with a reported remission rate of approximately 40%.^10^ Despite this unfavorable prognosis, antimicrobial therapy remains the primary and most effective treatment for IEAGN.

In our case, serum cryoglobulin levels became undetectable within 15 days of initiating antimicrobial therapy. Although there are no definitive studies detailing a timeline for cryoglobulin clearance, one study reported its disappearance within 6 weeks^9^ and another reported no recurrence of cryoglobulinemia over a 5-year period. These findings, along with our case, suggest that cryoglobulinemia associated with IEAGN often diminishes quickly with antimicrobial therapy and rarely recurs.

## Conclusions

Our report highlights a rare case of cryoglobulin-positive IEAGN. When cryoglobulinemia is identified in clinical practice, routine blood cultures and echocardiography are essential to rule out IE.

Cryoglobulin-positive IEAGN should be treated with conventional antimicrobials, with serum cryoglobulin levels typically returning to normal after appropriate treatment. Early identification and appropriate treatment are crucial for improving outcomes.

**Visual Summary:** Timeline of a Case: IEAGN With Cryoglobulin Positivity

Timeline	Events
Day 1	A woman in her 70s with a history of type 2 diabetes and mitral valve prolapse visited our hospital with a chief complaint of weight loss. She was initially referred to a diabetologist for follow-up care.
Day 23	Urinalysis revealed proteinuria (urine protein/creatinine ratio of 0.75 g/gCr) and hematuria. She was referred to the nephrology department.
Day 44	The patient’s estimated glomerular filtration rate (eGFR) declined rapidly from 58 to 27 mL/min/1.73 m^2^ in <30 d. To prevent further renal decline, treatment with 40 mg of prednisolone was initiated.
Day 52	A renal biopsy was performed, revealing pathologic evidence of endocapillary proliferative glomerulonephritis. The patient’s serum cryoglobulin level was positive. She was initially diagnosed with cryoglobulinemic glomerulonephritis due to essential type II mixed cryoglobulinemia.
Day 68	Prednisolone was reduced to 30 mg/d. Her renal function did not improve.
Day 73	The patient’s serum cryoglobulin level was positive.
Day 82	Prednisolone was reduced to 20 mg/d. Her renal function did not improve.
Day 83	The patient suddenly developed a middle cerebral artery infarction and exhibited signs of infection, raising suspicion of infective endocarditis (IE). Echocardiography revealed mitral valve vegetation, prompting a referral to a cardiologist for further evaluation and treatment.
Day 85	Three blood culture results revealed a gram-positive coccus called “*Granulicatella adiacens*.” A definitive diagnosis of IE was established using the modified Duke criteria. Benzylpenicillin potassium and ceftriaxone sodium hydrate were administered in combination.
Day 95	eGFR improved to 57 mL/min/1.73 m^2^.
Day 99	The patient’s serum cryoglobulin level became negative.
Day 119	The patient’s serum cryoglobulin level was negative. She was discharged and referred to a cardiovascular surgeon because a 10-mm vegetation remained attached to the mitral valve.

## Funding Support and Author Disclosures

The authors have reported that they have no relationships relevant to the contents of this paper to disclose.

## Appendix

Video 1 Previous Transthoracic Echocardiographic Images (2 Years Before Admission) (A and B) Mitral regurgitation at the color Doppler signal. (C and D) Transthoracic echocardiography reveals prolapse of the mitral valve posterior leaflets without vegetation.

Video 2 Transthoracic Echocardiographic Images on Admission (A and B) Mitral regurgitation at the color Doppler signal. (C and D) Transthoracic echocardiography reveals vegetation of the mitral valve posterior leaflets.
